# Sudden Cardiac Arrest in a Patient With Sarcoidosis and Familial Hypercholesterolemia

**DOI:** 10.7759/cureus.13649

**Published:** 2021-03-02

**Authors:** Hiroshi Sugimoto, Mariko Takeuchi, Yayoi Taniguchi, Junya Sato

**Affiliations:** 1 Department of Respiratory Medicine, Kobe Red Cross Hospital, Kobe, JPN; 2 Department of Cardiology, Kobe Red Cross Hospital, Kobe, JPN

**Keywords:** sarcoidosis, familial hypercholesterolemia, atherosclerosis, cardiac arrest, coronary artery disease

## Abstract

A 32-year-old Japanese man experienced out-of-hospital cardiac arrest. On arrival, computed tomography (CT) showed ground-glass opacity in the right lung. Emergency coronary angiography revealed triple vessel disease, then he underwent percutaneous coronary intervention. We also diagnosed him with heterozygous familial hypercholesterolemia and administered rosuvastatin and evolocumab. His clinical course was uncomplicated, and he was discharged on the 21st day of admission.

Follow-up CT performed two years later revealed multiple areas of consolidation with sarcoid galaxy sign and mediastinal lymphadenopathy. We diagnosed him with pulmonary sarcoidosis by histopathological evaluation of the biopsied specimen via endobronchial ultrasound-guided transbronchial fine-needle aspiration of enlarged subcarinal lymph nodes. After we administered oral prednisolone with a gradual taper, his CT findings improved.

## Introduction

Sarcoidosis is a systemic disease characterized by the formation of non-caseous granulomas secondary to unknown pathomechanisms. Most patients with sarcoidosis are asymptomatic or show a self-limited disease course; however, cardiac sarcoidosis can cause sudden death [1].

Familial hypercholesterolemia (FH) is an autosomal dominant disorder secondary to a mutation of the low-density lipoprotein (LDL) cholesterol receptor gene. Patients with FH, particularly homozygous FH, usually show elevated serum LDL cholesterol levels and are predisposed to ischemic heart disease associated with coronary atherosclerosis at an early age [2,3]. We report a case of sudden cardiac arrest in a patient with sarcoidosis and FH.

## Case presentation

A 32-year-old Japanese man with an unremarkable medical history (except for untreated hypercholesterolemia) developed out-of-hospital cardiac arrest while playing baseball. Cardiopulmonary resuscitation performed immediately by witnesses resulted in return of spontaneous circulation after a single defibrillation attempt via an automated external defibrillator, and he was transported to our hospital by the emergency medical service.

**Figure 1:**
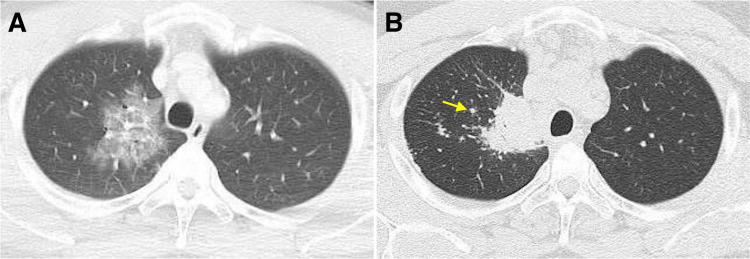
Non-contrast enhanced chest CT findings. (A) Chest CT showing a GGO in the right upper lobe. (B) Chest CT performed two years later showing the GGO appeared as a central core of coalescing granulomas, with peripheral nodules (arrow) reminiscent of a globular cluster or galaxy (sarcoid galaxy sign). CT: computed tomography, GGO: ground-glass opacity

Upon arrival at our hospital, contrast-enhanced whole-body computed tomography (CT) revealed a right upper lobe ground-glass opacity (GGO), without any other significant finding (Figure 1A).

Emergency coronary angiography revealed triple-vessel disease and 99% stenosis of the left anterior descending artery for which he underwent percutaneous coronary intervention (PCI). Blood test results showed elevated total serum cholesterol of 330 mg/dL and LDL cholesterol of 261 mg/dL. His Achilles tendon thickness was 16 mm, and he revealed a family history of FH in his biological mother. Therefore, we diagnosed him with heterozygous FH. Elevated serum LDL cholesterol levels persisted despite rosuvastatin treatment; therefore, we added evolocumab to his regimen. His post-PCI clinical course was uncomplicated, and he was discharged on the 21st day of admission.

Follow-up CT performed two years later revealed multiple areas of consolidation and mediastinal lymphadenopathy. The GGO in the right upper lobe appeared as a central core of coalescing granulomas, with peripheral nodules reminiscent of a globular cluster or galaxy (sarcoid galaxy sign, Figure 1B). We performed endobronchial ultrasound-guided transbronchial fine-needle aspiration of enlarged subcarinal lymph nodes. Histopathological evaluation of the biopsied specimen showed non-caseous granulomas, which confirmed the diagnosis of pulmonary sarcoidosis. Electrocardiography and transthoracic echocardiography revealed no specific findings of cardiac sarcoidosis, including atrioventricular block and basal thinning of the interventricular septum. We did not perform additional investigations for cardiac sarcoidosis, such as cardiac magnetic resonance imaging, positron emission tomography, and myocardial biopsy.

**Figure 2:**
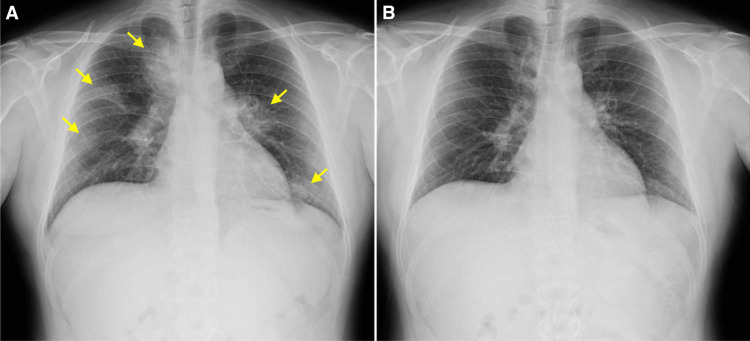
Chest x-ray findings before and after steroid therapy. (A) Chest x-ray taken prior to steroid therapy shows multiple consolidations (arrows) consistent with pulmonary sarcoidosis. (B) Chest x-ray shows multiple consolidations resolved after steroid therapy.

He had only chest discomfort but his imaging findings had progressively worsened; therefore, we administered oral prednisolone (40 mg) every other day with a gradual taper for pulmonary sarcoidosis. After the steroid treatment, the patient’s chest CT findings improved (Figures 2A, 2B), and his serum LDL cholesterol levels were still controlled below at most 100 mg/dL with rosuvastatin and evolocumab combination therapy.

## Discussion

It is well known that FH induces coronary artery disease, which can be a cause of sudden cardiac death [2]. In the present case, the onset of cardiac arrest was relatively young, but it is consistent with previous reports [2]. Although FH seems to be one of the most important contributors to coronary artery disease, we believe that pulmonary sarcoidosis also contributed to coronary atherosclerosis followed by sudden cardiac arrest.

Some clinical studies have reported poor cardiac outcomes in patients with sarcoidosis. Ekström et al. reported severe coronary artery disease in four of 38 (11%) autopsy cases in which cardiac sarcoidosis had led to sudden death [4]. Ungprasert et al. observed a high incidence of cardiovascular disease in patients with sarcoidosis [5]. In a large cohort study reported by Yafasova et al., patients diagnosed with sarcoidosis showed a high risk of heart failure and other adverse cardiac events [6]. However, whether patients with pulmonary sarcoidosis without cardiac involvement also show a high risk of poor cardiac outcomes remains unknown.

The pathomechanisms that explain the association between sarcoidosis and coronary artery disease remain unclear. However, recent research in lipidomics has proposed some hypotheses. For example, chitotriosidase (human chitinase 1) is considered an indicator of macrophage activity and may be an inflammatory pathomechanism shared by sarcoidosis and atherosclerosis [7].

Although further research is warranted to conclusively establish this association, we conclude that sarcoidosis may have accelerated coronary atherosclerosis secondary to FH in the present case. Notably, corticosteroids administered for sarcoidosis may cause adverse effects, including hypertension and dyslipidemia [8].

## Conclusions

In the present case, both sarcoidosis and FH can be the causes of cardiac sudden death. Recent studies in the field of lipidomics report that sarcoidosis and atherosclerosis share several inflammatory mechanisms that contribute to their pathogenesis. Clinicians should be aware of the potential risk of sarcoidosis in patients with atherosclerosis, particularly in those with concomitant risk factors such as FH.
